# Multimethod Assessment of Design, Metallurgical, and Mechanical Characteristics of Original and Counterfeit ProGlider Instruments

**DOI:** 10.3390/ma15113971

**Published:** 2022-06-02

**Authors:** Jorge N. R. Martins, Emmanuel J. N. L. Silva, Duarte Marques, Sofia Arantes-Oliveira, António Ginjeira, João Caramês, Francisco M. Braz Fernandes, Marco A. Versiani

**Affiliations:** 1 Faculdade de Medicina Dentária, Universidade de Lisboa, 1600-277 Lisboa, Portugal; duartemd@yahoo.co.uk (D.M.); sofiaaol@campus.ul.pt (S.A.-O.); aginjeira@campus.ul.pt (A.G.); carames@campus.ul.pt (J.C.); 2 Unidade de Investigação em Ciências Orais e Biomédicas (UICOB), Faculdade de Medicina Dentária, Universidade de Lisboa, 1600-277 Lisboa, Portugal; 3 Centro de Estudo de Medicina Dentária Baseada na Evidência (CEMDBE), Faculdade de Medicina Dentária, Universidade de Lisboa, 1600-277 Lisboa, Portugal; 4 Instituto de Implantologia, 1070-064 Lisboa, Portugal; 5 Department of Endodontics, School of Dentistry, Grande Rio University (UNIGRANRIO), Rio de Janeiro 21210-623, Rio de Janeiro, Brazil; nogueiraemmanuel@hotmail.com; 6 Department of Endodontics, Fluminense Federal University, Niteroi 24220-900, Rio de Janeiro, Brazil; 7 LIBPhys-FCT UID/FIS/04559/2013, 1600-277 Lisboa, Portugal; 8 BIOMAT, Unidade de Investigação em Ciências Orais e Biomédicas (UICOB), FMDUL, 1600-277 Lisboa, Portugal; 9 CENIMAT/I3N, Department of Materials Science, NOVA School of Science and Technology, Universidade NOVA de Lisboa, 2829-516 Caparica, Portugal; fbf@fct.unl.pt; 10 Dental Specialty Center, Brazilian Military Police, Belo Horizonte 30350-190, Minas Gerais, Brazil; marcoversiani@yahoo.com

**Keywords:** bending load, counterfeit, cyclic fatigue, design, differential scanning calorimetry, endodontics, torsional strength

## Abstract

A multimethod study was conducted to assess the differences between original (PG-OR) and counterfeit (PG-CF) ProGlider instruments regarding design, metallurgical features, and mechanical performance. Seventy PG-OR and PG-CF instruments (n = 35 per group) were evaluated regarding the number of spirals, helical angles, and measuring line position by stereomicroscopy, while blade symmetry, cross-section geometry, tip design, and surface were assessed by scanning electron microscopy. Energy-dispersive X-ray spectroscopy and differential scanning calorimetry were used to identify element ratio and phase transformation temperatures, while cyclic fatigue, torsional, and bending testing were employed to assess their mechanical performance. An unpaired t-test and nonparametric Mann–Whitney U test were used to compare instruments at a significance level of 5%. Similarities were observed in the number of spirals, helical angles, blade symmetry, cross-sectional geometries, and nickel–titanium ratios. Measuring lines were more reliable in the original instrument, while differences were noted in the geometry of the tips (sharper tip for the original and rounded for the counterfeit) and surface finishing with PG-CF presenting more surface irregularities. PG-OR showed significantly more time to fracture (118 s), a higher angle of rotation (440°), and a lower maximum bending load (146.3 gf) (*p* < 0.05) than PG-CF (*p* < 0.05); however, maximum torque was similar for both instruments (0.4 N.cm) (*p* > 0.05). Although the tested instruments had a similar design, the original ProGlider showed superior mechanical behavior. The results of counterfeit ProGlider instruments were unreliable and can be considered unsafe for glide path procedures.

## 1. Introduction

Glide path is defined as a clinical procedure to expand or create a smooth tunnel from the coronal portion of the root canal to the foramen, aiming to control the torsional stress and prevent breakage of nickel–titanium (NiTi) rotary instruments before the final canal enlargement [1]. This procedure is divided into two sequential steps: the micro glide path, usually performed with small-sized hand files for canal scouting and patency, and the macro glide path, using additional hand files or low-tapered mechanically driven NiTi instruments [2]. In the market, several rotary NiTi instruments have been designed to perform the macro glide path including R-Pilot (VDW, Munich, Germany), HyFlex GPF (Coltene, Allstetten, Switzerland), or ProGlider (Dentsply Sirona, Ballaigues, Switzerland). However, with the rise of new dental corporations in the major emerging economic countries manufacturing and marketing dental goods worldwide, a new phenomenon has been observed in recent years with the development of so-called replica-like and counterfeit instruments [3]. The former are manufactured by legalized companies and distributed worldwide under different brands, presenting characteristics that very closely mimic premium brands, while the latter are manufactured and packed to pass off as something that they are not, violating patent rights and being susceptible to legal and criminal punishment in some countries [4,5]. Recent studies have compared several replica-like and counterfeit instruments, showing that despite their overall design similarities, important differences that would impact their safety during clinical use are noted [3,5,6]. Independent of looking similar to genuine products, the counterfeit files have been linked to poorer performance in the scarce information available [3,5] and thus might also be considered to be a clinical risk for both dental practitioner and patient. Regarding glide path procedures, the use of replica-like or counterfeit NiTi instruments without scientific background regarding their efficacy and safety can be still more critical considering that they are used in narrow canals which tend to overstress the instrument during the root canal preparation procedure.

Two of the main concerns related to the use of NiTi rotary instruments are the possibility of file separation [7] and the occurrence of root canal preparation deviations due to the lack of the instrument’s flexibility [8]. In order to assess the file’s ability to bypass these concerns, multimethod research [6] has been advocated in order to determine the instrument’s mechanical strength at multiple tests and to correlate the results with multiple other instrument characteristics. This approach allows for a more comprehensive assessment of the instrument’s true characteristics.

Therefore, a multimethod study was conducted to assess the overall design, metallurgical properties, and mechanical performance of the original and counterfeit ProGlider instruments. The null hypothesis to be tested was that there are no differences between both instruments regarding their mechanical behavior.

## 2. Materials and Methods

**Figure 1:**
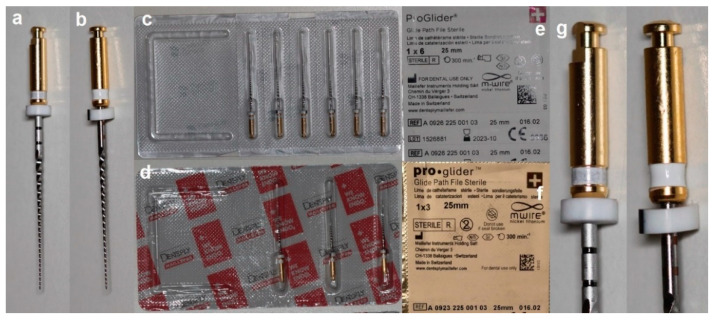
Macroscopic images, packing blisters, and labeling of (**a**,**c**,**e**) original (PG-OR) and (**b**,**d**,**f**) counterfeit (PG-CF) ProGlider instruments. On the right (**g**), enlarged images of the handles, measuring stops, and lines of the PG-OR (left) and PG-CF (right) instruments showing distinct size and color rings. Note that the measuring lines of PG-CF are painted or laser printed without relief.

Seventy original (PG-OR) and counterfeit (PG-CF) ProGlider instruments (35 per group) (Table 1 and Figure 1) were tested regarding geometric design, metallurgical properties, and mechanical performance.

### 2.1. Instruments’ Design

Instruments from each system (n = 6) were randomly selected and examined at ×3.4 and ×13.6 magnifications under stereomicroscopy (Opmi Pico, Carl Zeiss Surgical, Jena, Germany) to evaluate (a) the number of active blades (in units), (b) the helical angle by calculating the average angle of the 6 most coronal spirals assessed in triplicate, and (c) the distance (in mm) from the 2 measuring lines (20 and 22 mm) to the instruments’ tip using a digital caliper with a 0.01 mm resolution (Mitutoyo, Aurora, IL, USA). Measurements were made in triplicate and averaged with values higher than 0.1 mm from the reference line position considered significant and (d) presence of major defects or deformations (missed, twisted, or distorted blades). These same instruments were then evaluated under scanning electron microscopy (SEM) (S-2400, Hitachi, Tokyo, Japan) at ×100 and ×500 magnification regarding the symmetry of the spirals (symmetrical or asymmetrical), the geometry of the tip (active or non-active), the cross-sectional shape, and the presence of surface marks, deformations, or defects produced by the machining process.

### 2.2. Metallurgical Characterization

The metallurgical characteristics of the instruments and their semi-quantitative elemental constitution were evaluated by using differential scanning calorimetry (DSC) (DSC 204 F1 Phoenix; Netzsch-Gerätebau GmbH, Selb, Germany) and energy-dispersive X-ray spectroscopy (Bruker Quantax, Bruker Corporation, Billerica, MA, USA) with scanning-electron microscopy (S-2400, Hitachi) (EDS/SEM), respectively. Fragments acquired from the coronal active portion of 2 instruments (3 to 5 mm in length) from each system, weighing 7 to 10 mg, were evaluated in the DSC test according to the American Society for Testing and Materials guidelines [9]. For 2 min, each sample was exposed to a chemical bath composed of a mixture of 45% nitric acid, 30% distilled water, and 25% hydrofluoric and then mounted in an aluminum pan, with an empty pan serving as control. In each group, DSC test was performed twice to confirm the results. Thermal cycles were performed from 150 °C to −150 °C (cooling/heating rate: 10 K/min), under a gaseous nitrogen (N_2_) atmosphere, and transformation temperature charts created with dedicated software (Netzsch Proteus Thermal Analysis; Netzsch-Gerätebau GmbH, Selb, Germany). EDS/SEM analysis was performed on the surface (400 µm^2^) of 3 instruments of each type at a 25 mm distance (20 kV and 3.1 A) using software with ZAF correction (Systat Software Inc., San Jose, CA, USA).

### 2.3. Mechanical Tests

The mechanical behavior of instruments (cyclic fatigue, torsional and bending resistance tests) was performed at room temperature (20 °C) (PTN) after all instruments were inspected under stereomicroscopy (×13.6 magnification) and no deformation or defects were observed. The final sample size calculation has taken into consideration the 6 initial results of each test with an 80% power and a 5% alpha-type error. For the time to fracture, maximum torque, angle of rotation, and maximum load tests (effect sizes of 84.2 ± 45.4, 0.05 ± 0.13, 66.8 ± 44.3, and 98.9 ± 53.4, respectively), a total of 6, 107, 8, and 6 instruments per group were determined, respectively. Then, a final sample size was set at 8 instruments per group for each test. For the cyclic fatigue test, a non-tapered custom-made stainless-steel tube apparatus was used [3,10] with instruments activated at a static position using a 6:1 reduction handpiece (Sirona Dental Systems GmbH, Bensheim, Germany), in a continuous rotary motion (300 rpm, 3.5 N.cm), powered by a torque-controlled motor (VDW Silver; VDW GmbH, Munich, Germany) using glycerin as lubricant. The files were able to rotate freely on a canal with 86 degrees of curvature and 6 mm curvature radius, which had a 9 mm length with the point of maximum stress load positioned in the middle of the curvature length. Time to fracture (in seconds) was set when fracture was detected by visual and auditory inspection, while the fragment size (in mm) was recorded for experimental control. Torsional and bending resistance tests were performed following international specifications [11,12]. To calculate the maximum torque (in N.cm) and angle of rotation (in degrees) prior to fracture, instruments were clamped in their apical 3 mm and rotated clockwise on a constant pace (2 rotations/min) until rupture (TT100 Odeme Dental Research, Luzerna, Santa Catarina, Brazil). For testing the maximum bending load for a 45° displacement (in gram/force; gf) using a load of 20 N and 15 mm/min of constant speed, instruments were mounted in the file holder of a motor and positioned at 45° in relation to the floor, while their apical 3 mm were attached to a wire connected to a universal testing machine (Instron EMIC DL-200 MF, São José dos Pinhais, Brazil).

### 2.4. Statistical Analysis

Statistical testing of normality of data distribution was performed using the Shapiro–Wilk test. Fragment length and angle of rotation were compared using unpaired *t*-test, while nonparametric Mann–Whitney U test was selected to evaluate time to fracture, maximum torque, and maximum bending load. Results were summarized using mean (standard deviation) and median (interquartile range) values at a significance level of 5% (SPSS v22.0 for Windows; SPSS Inc., Chicago, IL, USA)

## 3. Results

### 3.1. Instruments’ Design

**Figure 2:**
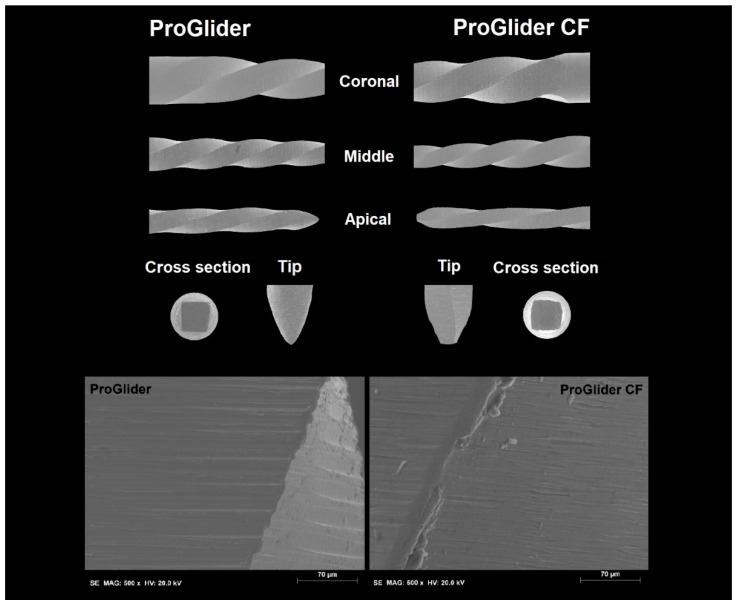
Representative SEM images of the coronal, middle, and apical portions of the active blades (on top) and cross-section and tip geometry (on the middle) of the original (PG-OR) and counterfeit (PG-CF) ProGlider instruments. Both instruments have symmetrical blade geometry without radial land and a square cross-sectional design. Differences can be seen in their tips, with PG-CF having a sharper tip and PG-OR a rounded one. The surface finishing analysis of both instruments (on the bottom) revealed parallel marks compatible with the manufacturing process; however, PG-CF showed more irregularities in its blade edges.

PG-OR and PG-CF had the same number of blades, similar helical angles, and an absence of major deformations, but the measuring lines of PG-CF were 0.7 mm above the reference value (Table 2). Moreover, PG-CF had distinct color-coding white rings and a measuring marks printing design compared to the PG-OR (Figure 1). SEM analysis of both instruments showed a symmetrical blade geometry without radial land and a square cross-sectional design, while clear differences were noted in their tips, with PG-OR having a sharper tip and PG-CF a rounded one (Figure 2). The surface finishing analysis revealed grinding manufacturing marks in both instruments; however, PG-CF showed additional irregularities and microdefects, such as metal rollovers, in its blade edges (Figure 2).

### 3.2. Metallurgical Characterization

**Figure 3:**
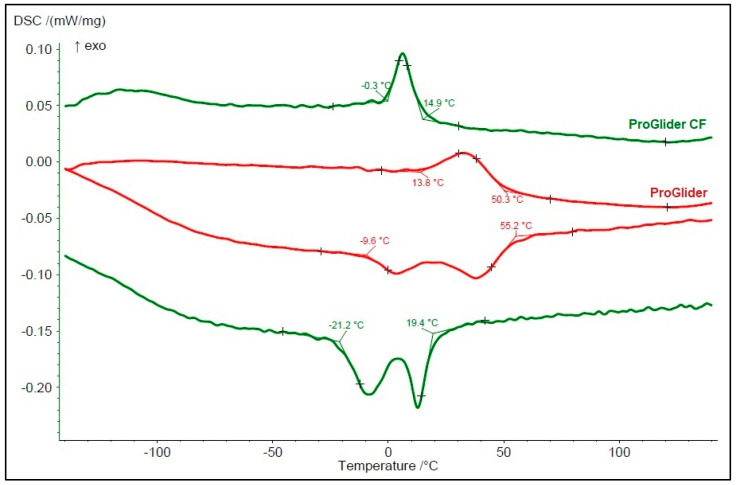
DSC chart showing the cooling curves on the top (right to left direction) and the heating curves on the bottom (left to right direction) of original (PG-OR in red) and counterfeit (PG-CF in green) ProGlider instruments. The test demonstrated that PG-CF is fully austenitic (R-phase start 14.9 °C), while PG-OR instrument was mixed austenite plus R-phase (R-phase start and finish were 50.3 °C and 13.8 °C, respectively).

In the EDS test, the alloy of both instruments showed an almost equiatomic relation between a nickel and titanium element (Ni/Ti ratio 1.017 [PG-OR] and 1.024 [PG-CF]), without traces of any other metal. The DSC test revealed the presence of heat treatment in both instruments (more notorious in PG-CF); however, while the PG-OR showed a mixed austenite plus R-phase constitution at room temperature (20 °C), PG-CF was fully austenitic. The R-phase start (Rs) and finish (Rf) temperatures were 50.3 °C and 13.8 °C for PG-OR and 14.9 °C and −0.3 °C for PG-CF, respectively (Figure 3).

### 3.3. Mechanical Tests

PG-OR showed higher mean time to fracture (118.0 s) compared to PG-CF (34.1 s) (*p* < 0.05), with no significant differences detected in their fragment length (*p* > 0.05) (Table 3). In the torsional test, similar mean maximum torques were observed for both instruments (0.4 N.cm), but the PG-OR showed the highest angle of rotation (440°) (*p* < 0.05) (Table 3). In the maximum bending load test, PG-OR was significantly more flexible (146.3 gf) than PG-CF (246.5 gf) (*p* < 0.05) (Table 3).

## 4. Discussion

The present study presents original and innovative results comparing original (PG-OR) and counterfeit (PG-CF) ProGlider instruments. The latter was acquired from an online shop (aliexpress.com) for 1/3 of the original brand price (Table 1) and further confirmed as counterfeit by Dentsply. The overall low prices of counterfeit and replica-like rotary instruments can be considered by some clinicians as a viable alternative to the original brands in order to minimize costs, as previously reported [13]. However, these products have already been associated with lower quality and mechanical behavior [3,5] compared to their respective premium brands. Therefore, considering the exponential growth of these products on a global scale, sequential studies must be done to minimize, or even deter, their use, protecting the original brand trademarks, patents, clinicians, and patients.

In the present study, although similarities could be observed regarding the number of blades, helical angle (Table 1), blade geometry, cross-sectional shape (Figure 2), and NiTi ratio elements (EDS test), differences between PG-OR and PG-CF were very clear starting from basic qualitative aspects, such as the identification of the instruments, which included large discrepancies in the dimensions of the white rings and measuring line position (Figure 1, Table 1). These differences were also noted in a previous study comparing original and counterfeit Reciproc (VDW, Munich, Germany) instruments, strengthening the conviction that counterfeit NiTi instruments are not made to match the original brands exactly. However, these are not relevant parameters if they do not impact the mechanical behavior and safety of the instruments. Thereby, a multimethod approach [10] was used in this study considering that it has been considered to be the most effective and reliable manner to perform a complete and comprehensive assessment of the multiple features and characteristics of the instruments, taking advantage of each methodology’s strengths [14]. Although both instruments were made of a NiTi alloy with an almost equiatomic NiTi ratio (EDS analysis), significant differences were observed in the phase transformation temperatures, with PG-CF being fully austenitic at room temperature (test temperature) and PG-OR having a mixed austenite plus R-phase (Figure 3). Considering the similarities in the instruments’ design and Ni-Ti ratio, differences in the surface finishing (Figure 2) and phase transformation temperatures (DSC analysis) are the parameters to take into consideration to explain the differences observed in the mechanical tests (Table 3).

Overall, although similar results were observed in maximum torque, all other mechanical parameters presented differences among the instruments (Table 3), and therefore the null hypothesis was rejected. Cyclic fatigue is a common test used to show the ability of NiTi instruments to sustain stress during flexion while in rotation around a predefined curvature [15], a reference value to which to compare the endurance of the instruments when shaping a curved canal. The time to fracture of PG-OR was 3.4 times higher (118.0 s) than PG-CF (34.1 s). This difference can be easily explained not only by the irregular surface of PG-CF, which may serve as stress points that may lead to crack initiation [16], but also by its austenitic nature, which tends to reduce the time to fracture when compared to R-Phase instruments [17,18]. The torsional test has been used to assess the capacity of an instrument to sustain a twisting axial force [19], with the maximum torque referring to the maximum load an instrument is able to sustain when twisted and the angle of rotation representing the maximum deformation an instrument is able to sustain before fracture. The ability to sustain twisting stress is of utmost importance during the mechanical action of cutting dentin, especially in narrow canals. Instruments made of austenitic NiTi alloy tend to present higher torsional strength then non-austenitic instruments [20,21]. However, this was not observed in this study, and PG-CF had a similar maximum torque and lower angle of rotation than PG-OR, which could be partially explained by its irregular blade edges and microdefects on its surface [16], ending up counterbalancing the expected result. Finally, the flexibility of an instruments can be assessed by the bending testing. This property is considered important in order to preserve the original path when shaping a curved canal. In this study, the austenitic nature of the counterfeit instrument explains its lowest flexibility.

One of the main strengths of the present study was that it consisted of multimethod research following widely accepted guidelines for DSC [9], torsional resistance, and the bending test [11,12]. Additionally, although some debate still exists regarding the parameters of cyclic fatigue tests [22], a well-established methodology was followed in this study [3,10]. In brief, this method uses a static position of the handpiece, which has been considered more reliable than the dynamic mode [22], and a non-tapered artificial canal, in which comparable ranking results could be expected in tapered canals, as long as the independent variables are the instruments, and not the artificial canals. Finally, room temperature was used instead of body temperature in the cyclic fatigue test because (i) the short contact time of the instrument with the dentinal walls does not apparently change the surface temperature of the instrument to reach body temperature [23], (ii) irrigant solutions are often used at room temperature, (iii) dentin-insulating efficiency prevents the instrument from reaching body temperature in clinics [24], (iv) instruments are sold and stored at room temperature, and (v) the manufacturer of the ProGlider does not recommend heating the instrument prior to or during its use. On the other hand, this study also presents limitations, considering the lack of tests involving dentin, such as cutting efficiency or shaping ability, which would give additional information regarding the efficiency and safety of the instruments. Although these supplementary tests might be seen as options for further research, it is important to highlight that the tests presently conducted demonstrated that the premium brand outperformed the mechanical behavior of the counterfeit version, which proved to be more prone to an early fracture and, due to its greater rigidity, may tend to deviate more easily from the original root canal trajectory in curved roots.

## 5. Conclusions

Overall, PG-OR outperformed its counterfeit version in cyclic fatigue testing (118.0 s and 34.1 s, respectively), while showing a higher flexibility with a higher angle of rotation (440.0° and 361.3°) and lower maximum bending load (146.3 gf and 246.7 gf). Additionally, PG-CF showed irregular blade edges, microdefects, and different phase transformation temperatures compared to the premium brand instrument. Results of PG-CF were unreliable, and this instrument can be considered unsafe for glide path procedures.

## Figures and Tables

**Table 1:** Characteristics of the original (PG-OR) and counterfeit (PG-CF) ProGlider instruments.

System	Metal Alloy	Category	Manufacturer Specifications	Identification (Color Coding)	Acquisition (Country)	Lot	Reference Price **
PG-OR	M-Wire	Premium brand	Dentsply (Ballaigues, Switzerland)	White ring	Local market (Portugal)	1526881	1.00
PG-CF *	M-Wire	Counterfeit	Dentsply (Ballaigues, Switzerland)	White ring	Internet (China)	1370977	0.26

* These instruments were confirmed as counterfeit by the original manufacturer (Dentsply). Therefore, information regarding metal alloy, manufacturer specifications, and lot number depicted on their label cannot be confirmed as real; ** Reference price value of the counterfeit instrument was rated based on the reference price of the premium brand instrument categorized as 1.

**Table 2:** Stereomicroscopic assessment of the original (PG-OR) and counterfeit (PG-CF) ProGlider instruments (median and interquartile range).

System	n	Number of Blades	Helical Angle (in °)	Measuring Lines Position (in mm)		Defects or Deformations
				20 mm	22 mm	
PG-OR	6	21	21.4 [20.6–21.9]	19.9 [19.7–20.0]	22.0 [21.9–22.1]	0
PG-CF	6	21	21.9 [20.9–22.7]	**20.7 [20.6–20.9]**	**22.7 [22.6–22.8]**	0

Significant discrepancies (values higher than 0.1 mm from the reference value) in the measuring line position were identified with bold letters.

**Table 3:** Mean (standard deviation) and median (interquartile range) results of the mechanical tests of the original (PG-OR) and counterfeit (PG-CF) ProGlider instruments.

System	Cyclic Fatigue Test		Torsional Test		Bending Test
	Time to Fracture (in Seconds)	Fragment Length (in mm)	Maximum Torque (in N·cm)	Angle of Rotation (in °)	Maximum Load (in gf)
PG-OR	118.0 (±13.7) 123.5 [106.5–128.5]	6.9 (±0.2) 7.0 [6.9–7.1]	0.4 (±0.1) 0.4 [0.3–0.5]	440.0 (±27.5) 439.0 [413.3–467.0]	146.3 (±11.5) 145.9 [141.7–150.9]
PG-CF	34.1 (±5.4) 34.0 [30.3–36.5]	6.8 (±0.7) 6.8 [6.2–7.6]	0.4 (±0.1) 0.4 [0.3–0.5]	361.3 (±37.2) 361.5 [329.0–400.0]	246.7 (±17.7) 246.5 [239.0–262.6]
*p*-value	<0.001	0.627	0.798	<0.001	<0.001
